# Moving Beyond Barker’s Hypothesis Towards New Therapies for the Treatment of Fetal Growth Restriction

**DOI:** 10.1093/function/zqaf022

**Published:** 2025-05-29

**Authors:** Laura E Coats, Barbara T Alexander

**Affiliations:** Department of Obstetrics and Gynecology, University of Mississippi Medical Center, Jackson, MS 39216, USA; Department of Physiology and Biophysics, University of Mississippi Medical Center, Jackson, MS 39216, USA

Fetal growth restriction (FGR) affects 5%-10% of all pregnancies and occurs when the fetus does not reach its full growth potential *in utero*.^1^ FGR is associated with an increased risk for perinatal morbidity and mortality and is a common cause of preterm birth.^1^ First postulated by David Barker, it is now well established that FGR is also associated with enhanced risk for cardiovascular and metabolic dysfunction throughout the lifespan,^2^ suggesting that FGR not only impacts fetal health and development, but also programs long-term effects that extend well beyond birth. The etiology of FGR can be due to a multitude of maternal factors, including pre-eclampsia, undernutrition, smoking, substance abuse, infections, or autoimmune diseases; placental dysfunction, such as placental insufficiency or placental abruption, or fetal conditions that include metabolic and genetic factors can also contribute to the development of FGR.^1^ Yet, a definitive cure for FGR remains elusive, and currently available treatment options involve management of the underlying cause(s) of FGR and preterm delivery.^1^ However, preterm delivery further promotes morbidity and mortality risk to the already compromised neonate.^1^ Thus, it is imperative to identify FGR in early gestation and, importantly, address a major gap in knowledge, the identification and development of therapeutic approaches that will improve fetal growth and increase the length of gestation.

To date, numerous interventions have been investigated as potential therapeutics for the treatment of FGR. Improvement of nitric oxide availability through the use of a phosphodiesterase type 5 inhibitor such as sildenafil citrate has been extensively researched in pregnancies complicated by pre-eclampsia, preterm labor, and FGR. Yet, a meta-analysis of clinical use of sildenafil for the treatment of FGR showed limited to no improvement in birthweight or gestational age at delivery.^3^ Survival and perinatal morbidity were also not improved, and the Dutch arm of the study was ended prematurely due to increased neonatal mortality and the development of newborn persistent pulmonary hypertension.^4^ Low-dose aspirin (60-150 mg/day) before 16 weeks of gestation in high-risk pregnancies is recommended and shows optimal maternal benefits. Although low-dose aspirin prophylaxis in high-risk pregnancies involves assessment for FGR, FGR is typically a secondary outcome and the benefit on fetal growth is unclear.^5^ Low-molecular-weight heparin, when prescribed at less than 16 weeks of gestation, is also associated with improved placental-mediated complications and, in some instances, improved fetal growth; however, these findings have not consistently demonstrated a benefit in the prevention of FGR.^5^ Other approaches using liposomes, adenoviral vectors, adeno-associated virus delivery, and polymer nanoparticles that target the uterine arterial endothelium or trophoblasts also show pre-clinical promise as potential therapeutic approaches.^6^ For example, pre-clinical use of gene therapy to target the maternal uterine artery circulation with vascular endothelial growth factor, a key regulator of placental cell growth and proliferation, demonstrates improved uterine blood flow, an increase in fetal weight with no vector spread to the fetus, or adverse effects on the fetus or mother.^7^ Although pre-clinical studies utilizing targeted approaches are identifying new opportunities for the treatment of many diseases, including FGR, translation to the clinical setting for the treatment of pregnancy-related diseases is still in development. Moreover, the etiology of FGR is multifactorial, and interventions that target uterine blood flow to improve fetal growth often lack consideration of the importance of placental function in the development of novel approaches for the treatment of FGR.

In this issue of *Function*, Karmer et al. explore placental nutrient transport pathways as a potential therapeutic target in the mitigation of FGR.^8^ Appropriate fetal growth involves placental transport of glucose, amino acids, and fatty acids to the developing fetus, and placental amino acid transporters, Systems A and L, are two of the most extensively studied placental nutrient transporters in human and non-human species. Placental insulin/insulin-like growth factor-1 (IGF-1) and mechanistic target of rapamycin (mTOR) are positively correlated with FGR and placental System A amino acid transporter expression in obese women who give birth to large babies,^9^ suggesting a critical role for placental signaling and up-regulation of amino acid transporters in fetal overgrowth. Domain-containing mTOR-interacting protein (DEPTOR) is an endogenous inhibitor of both mTOR complex 1 (mTORC1) and mTORC2 signaling. In this issue of *Function*, Karmer et al. demonstrated that placental DEPTOR protein expression was elevated in human FGR and that using a lentivirus-mediated trophoblast-specific knockdown of placental DEPTOR in mice, placental mTORC1 and mTORC2 signaling were activated, System A and L activity were increased, and importantly, fetal growth was improved.^8^ Collectively, findings from this study provide proof of principle that targeting specific upstream regulators of placental amino acid transporters serves as a novel approach to alleviate impaired fetal growth. In addition to Systems A and L, IGF is also associated with the regulation of glucose uptake in the placenta. Nanoparticle-mediated gene therapy targeting placental IGF-1 maintains fetal weight in a mouse model of FGR via an increase in glucose and amino acid transport,^6^ further suggesting that targeting placental nutrient transporters may serve as a critical interventional target for FGR and facilitate improvement in perinatal and long-term health outcomes. Yet, caveats persist. Lentivirus vectors are currently approved for genetic diseases and some cancers. However, translating lentiviral approaches in gene therapy for use in pregnancy is not yet approved. Moreover, approaches that specifically target placental pathways and regulate fetal growth must include careful consideration to not induce additional harm to the mother, the placenta, or the fetus. Placental nutrient transport is a complex and highly regulated process that is critical for proper fetal development and growth. Downregulation of placental System A amino acid transport occurs prior to the development of FGR in pregnancies complicated by undernutrition, suggesting that the reduction in placental transporter expression is a cause, not a consequence of FGR.^10^ However, per Barker’s original hypothesis of fetal undernutrition associated with brain sparing,^2^ fatty acid transporter expression and placental storage are increased in pregnancies complicated by placental insufficiency via a compensatory mechanism to maintain fetal brain growth and development. Collectively, these studies indicate that placental nutrient transport to the fetus in FGR is differentially regulated, emphasizing the caveat that potential therapeutic approaches for the mitigation of FGR should be specifically designed to maintain normal placental metabolism and fetal growth in fetuses with diagnosed FGR.
